# Recurrent Macroprolactinoma with Malignant Conversion to Carcinoma with Spinal Metastasis

**DOI:** 10.1155/2021/7488236

**Published:** 2021-11-11

**Authors:** Madeline Mori, Amanda Frugoli, Udesh Shah, Brad Barrows, Tricia Westhoff, David Westra

**Affiliations:** ^1^Western University of Health Sciences, Pomona, CA 91766, USA; ^2^Department of Graduate Medical Education, Community Memorial Hospital, Ventura, CA 93003, USA; ^3^Department of Internal Medicine, Community Memorial Hospital, Ventura, CA 93003, USA; ^4^Department of Pathology, Community Memorial Hospital, Ventura, CA 93003, USA; ^5^Department of Endocrinology, Community Memorial Hospital, Ventura, CA 93003, USA; ^6^Department of Neurosurgery, Community Memorial Hospital, Ventura, CA 93003, USA

## Abstract

In contrast to pituitary adenomas, pituitary carcinomas represent a rare malignant neoplasm with a remarkable high mortality. Pituitary carcinomas can arise from any pituitary tumor cell line and are determined to be carcinomas when there is distant metastasis or central nervous system dissemination. In this case vignette, we describe a rare case of malignant prolactinoma with intraspinal metastasis, and we also provide a review of relevant literature and treatment.

## 1. Introduction

Anterior pituitary adenomas/tumors (pituitary tumors) are not uncommon [1, 2]. In a meta-analysis of postmortem studies by Ezzat et al., pituitary tumors were incidentally found to have an estimated prevalence of 14.4% [1]. These tumors can also be incidentally detected by neuroimaging studies in up to 22.5% of the general population [1]. Pituitary tumors have traditionally been classified as adenomas (macroadenoma >1 cm; microadenoma <1 cm) but represent a diverse group of histologic subtypes [3]. To address the variability in clinical outcomes for these neoplasms, a new terminology, pituitary neuroendocrine tumors (PitNETs), has recently been proposed [4]. This proposal is based on the similar absence of specific histologic characteristics that could be used to reliably predict tumor behavior and is similar to the classification utilized in gastrointestinal neuroendocrine tumors. The current classification of pituitary tumors is primarily based on clinical findings (radiology, hormone-associated symptoms, and mass effect), histomorphology, and immunohistochemistry, with additional tools including ultrastructure analysis by electron microscopy and in difficult cases molecular studies [4, 5]. Nonfunctioning adenomas cause disease by slow growth and encroachment with displacement of brain structures (mass effect), while functioning adenomas produce symptoms related to hormone production [3, 6]. Despite the relatively high prevalence of pituitary tumors, only a small fraction (∼0.2%) undergoes malignant transformation to pituitary carcinoma, which has a remarkably high mortality [3, 4, 6]. Pituitary carcinomas can arise from any anterior pituitary tumor cell line and are defined when there is distant metastasis or central nervous dissemination [6–8]. Current limitations in the diagnosis and management of pituitary carcinomas arise from an inability to distinguish the potential for a pituitary adenoma to transform into a pituitary carcinoma based on histologic characteristics alone in most cases. It is only after metastasis is confirmed that a pituitary tumor can be classified as a pituitary carcinoma [9]. Pituitary carcinoma metastasis is commonly found in both intracranial (cerebral cortex, cerebellum, cerebellopontine, angle, and dura) and extracranial (lymph nodes, bones, lungs, and liver) locations [10–15]. Metastatic foci are typically identified by either manifestation of symptoms or as an incidental finding on imaging [11, 16].

Pituitary carcinomas comprise 0.2% of all pituitary tumors [17, 18]. Of the limited cases described, a large majority are hormonally active, with estimates approaching 88% [17, 19]. In Ragel and Couldwell's literature review, they were able to estimate that the majority of hormonally active carcinomas included ACTH at about 42% followed by prolactin producing 33%, followed by growth hormone, LF/FSH, and finally TSH producing [17]. Curiously, pituitary carcinomas have displayed a greater tendency toward systemic metastasis than craniospinal metastasis [3, 4, 19]. Despite the higher prevalence of ACTH hormone producing pituitary carcinomas, the rate of systemic metastasis is seen more commonly with prolactin producing tumors at 71% [19]. In a comprehensive review of 72 cases of pituitary carcinoma by Yoo et al., they found an average age of diagnosis of pituitary carcinoma to be 46.3 years, with a range from 9 to 75 years [20]. Diagnosis of pituitary carcinoma is “extremely rare” in children [21]. Of the 72 cases of pituitary carcinoma described by Yoo et al., there was only one pediatric case, a 9-year-old female with spinal and intracranial metastasis [20, 22].

Pituitary carcinomas are difficult to treat, with patients having an average of 2-3 surgeries in addition to radiation or chemotherapy treatment [20]. These carcinomas account for a striking mortality rate between 43% and 54.8% with a median survival of 10 months after diagnosis [13, 20]. In this case vignette, we describe a rare case of metastatic prolactinoma, and we also provide relevant literature review.

## 2. Case Report

The patient is a 71-year-old right-handed and physically active female with an extensive history of pituitary macroprolactinoma first diagnosed in 2006. Her treatment course has been complicated with recurrence and has required two resections. Initial treatment for her macroprolactinoma included a debulking procedure in September 2006, followed by partial transnasal transsphenoidal surgery in November 2012, and gamma knife external beam radiation treatment localized to the cavernous sinus in January 2014. She underwent radiographic and hormonal surveillance with MRI in August 2014 and July 2019. She concurrently underwent hormonal treatment with cabergoline. Pituitary MRI on August 2014 showed resolution of the tumor in the cavernous sinus. Pituitary MRI on November 2019 showed no evidence of tumor. She had normal prolactin levels between November 2014 and August 2017, with a steady increase from November 2017–July 2020 from 21.74 ng/ml to 656.60 ng/ml despite increasing the dose of cabergoline to 2 mg oral daily and a repeat course of gamma knife external beam radiation to the cavernous sinus in 2019.

**Figure 1:**
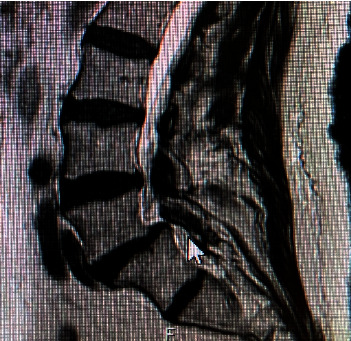
MRI lumbar spine with contrast showing grade 1 spondylolisthesis at L5–S1 with S1-S2 disk space causing severe spinal stenosis. There is a large object extending inferiorly from L5–S1 disk space.

**Figure 2:**
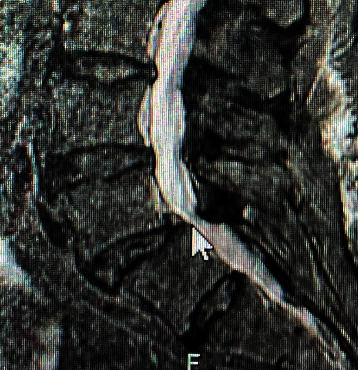
MRI lumbar spine with contrast showing an intradural and enhancing mass in the L5–S1 disc space causing severe stenosis within the intradural space. This object is an intradural enhancing mass causing severe stenosis within the intradural space.

She presented in August 2020 with constant sharp, burning 9/10 low back pain, and left lower extremity pain worsening for the past 2 months. She described her presenting pain as most intense in the left buttock, posterior thigh, and calf. Pain was aggravated by motion initially, with only slight relief by taking naproxen or ibuprofen. She denied weakness, numbness, bowel, or bladder difficulties. She was treated with physical therapy, with only slight benefit, and had otherwise not had any other treatment for her pain. On her neurologic exam, her sensation was intact to light touch and pinprick in all dermatomes except a slight decrease in pinprick sensation to both modalities at the lateral aspect of her left foot. Her reflexes were 2+ throughout except for absent left Achilles' jerk. No evidence of Hoffmann sign, Babinski sign, or clonus. Positive straight leg raise on the left side. She had an antalgic gait, but was able to walk with her heels, toes, and heel-to-toe. She was sent for neuroimaging of the lumbar spine (Figures 1 and 2).

**Figure 3:**
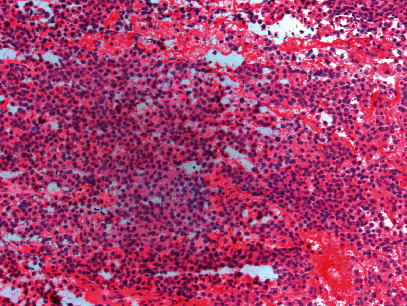
Intraoperative frozen section evaluation showing a homogeneous population of tumor cells with round nuclei, indistinct nucleoli, and cytoplasmic granules (200x magnification).

**Figure 4:**
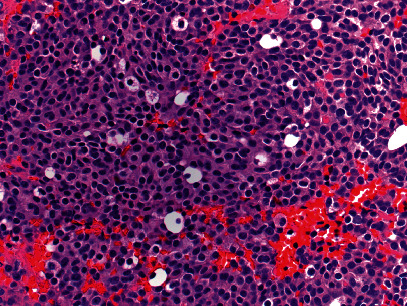
Permanent pathology demonstrating minimal nuclear pleomorphism and only rare mitotic figures (400x magnification).

**Figure 5:**
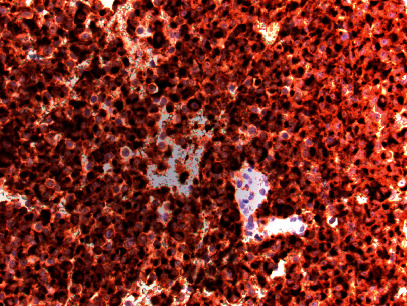
Immunohistochemical stain for prolactin showing prominent granular cytoplasmic staining with perinuclear Golgi pattern accentuation (400x magnification).

**Figure 6:**
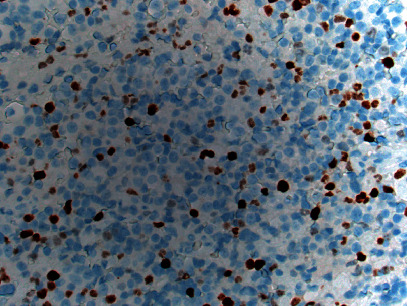
Immunohistochemical stain for Ki-67 showing a proliferation index of 18% by 1000 tumor cell count (400x magnification).

In August 2020, she was admitted to the hospital for surgical resection of an intradural tumor causing severe debilitating spinal stenosis (Figure 2). She underwent posterior lumbar laminectomy, facetectomy, and foraminotomy from L4 to S1 with instrumentation arthrodesis from L4 to S1. In addition to resection of the intradural tumor, she required posterolateral fusion of L4 and L5 with a partial reduction of L4-5 spondylolisthesis and use of an autograft. Intraoperatively, she was found to have a 3 cm friable tumor with nerve roots in the tumor capsule, which was highly suspicious for pituitary carcinoma given her history. Intraoperative frozen section pathology showed histologic features compatible with pituitary neoplasm (Figure 3). Permanent pathology showed minimal nuclear pleomorphism of tumor cells and overall histomorphology similar to a typical pituitary adenoma (Figure 4). Immunohistochemistry confirmed the presence of prolactin in tumor cells (Figure 5) and showed a moderately increased Ki-67 (Figure 6) proliferation index (18%; pituitary adenoma usually <3%). Given the location within the lumbar spine, the tumor met criteria for the final diagnosis of pituitary carcinoma, a lactotroph subtype associated with intradural, extramedullary drop metastasis. Postoperative imaging showed gross total resection of the tumor. Patient noted improved lower back pain and was evaluated by physical therapy and occupational therapy and was stable on discharge home hospital day 6. She had close follow-up labs in September 2020, November 2020, and March 2021 with reduced prolactin levels of 3.00, 1.23, and 1.17, respectively. She underwent 29 days of radiation therapy to the lumbosacral spine, which she completed in April 2021. She continues on cabergoline 2.0 mg daily and clinically is doing well.

## 3. Discussion

Pituitary tumors are characterized as functional or nonfunctional, i.e., hormone producing or nonhormone producing. Prolactinomas (prolactin secreting pituitary tumors) are the most commonly diagnosed hormone-producing pituitary adenomas, comprising about 40% of all functional pituitary tumors [2, 5]. Though pituitary adenomas are generally rare in children, of the pituitary adenomas, prolactinomas are the most common adenoma in children [21]. Many prolactinomas are asymptomatic and found incidentally on imaging or during autopsy [1, 2, 23]. Due to the inhibitory effect that prolactin has on follicle stimulating hormone and luteinizing hormone secretion, patients with functional prolactinomas may complain of symptoms typical of hypogonadism including decreased libido and fertility issues [2, 5]. Prolactinomas can also be classified based on size: <1 cm considered microprolactinoma and >1 cm considered macroprolactinoma [23]. Most prolactinomas (90%) are small and stay within the suprasellar space [5]. However, macroprolactinomas, defined as being more than 1 cm in size, often cause symptoms due to mass effect, commonly causing symptoms of visual field defects due to compression of the optic chiasm and headaches [5]. Depending on their size and functionality, they can be successfully treated with dopamine agonists, cabergoline or bromocriptine, or with resection [3].

A small subset of pituitary adenomas, 5%, show aggressive features, which are more difficult to manage, leading to increased mortality and morbidity [4, 6, 14]. Unlike other pituitary adenomas, aggressive adenomas tend to have multiple local recurrences or are found to be resistant to treatment [11, 17, 18]. Although there is no clear definition for an aggressive adenoma, Ilie et al. suggest thinking about those tumors that show clinically relevant tumor growth, for example, those causing signs and symptoms [6]. Although many aggressive adenomas have nuclear atypia and cellular pleomorphism, many studies have shown that histomorphology and/or immunostaining alone is not a good predictor of aggressive adenoma or potential for metastasis [8, 19, 20]. Weirinckz et al. specifically tried to evaluate diagnostic markers for prolactin pituitary tumors. In their review of proliferation markers, no marker could distinguish between noninvasive and invasive tumors. They were able to notice a small difference in mitosis and Ki-67 labeling, and they suggested using a combination of markers to reclassify adenomas as “atypical” [24]. This was introduced into the 2004 WHO classification of tumors, adding this focus of molecular biology and pathogenesis. However, it is not overwhelmingly supported as many authors have been able to demonstrate a lack of reliable histological markers to determine atypia (aggressive local behavior) or risk of developing carcinoma [16, 24]. These markers are likely limited in usefulness as more information is needed to determine the complex function that regulates pituitary plasticity and the role in normal physiological function vs. tumor behavior [24]. This is also limited by the multiple cell lines within the anterior pituitary and numerous possible combinations of histomorphology and immunostaining that may be unique to each cell line. Even more rarely, pituitary carcinomas consist of 0.2% of all pituitary tumors [18, 25]. Most authors believe that pituitary carcinomas evolve through malignant transformation from a preexisting adenoma rather than developing de novo, as both aggressive adenomas and pituitary carcinomas often show increased proliferation of Ki-67, increased mitosis, and/or p53 overexpression [7, 13, 24]. Most pituitary carcinomas are diagnosed between 4.7 and 9.04 years after initial pituitary tumor detection [7, 20].

Clinicians must have a high index of suspicion when managing a patient that has multiple occurrences after initial pituitary surgery [7, 20]. On average, patients with pituitary carcinoma had 2.77 surgical interventions before the diagnosis was confirmed [14]. Pituitary carcinoma with metastasis must be suspected in patients when there is a discrepancy between prolactin levels and MRI neuroimaging [5, 7]. Dedicated endocrine tumor imaging may be more helpful in these cases than standard 18F-FDG PET/CT and enhanced magnetic resonance imaging (MRI). Utilization of gallium-68 (68 Ga)-DOTATATE positron emission tomography (PET)/computer tomography (CT) may be more useful for evaluating metastatic CNS involvement [4, 11].

The European Society of Endocrinology (ESE) recently published clinical practice guidelines for the management of these forms of pituitary lesions, including aggressive pituitary tumors and carcinomas. They outline that optimal conventional medical and surgical treatment should be utilized as the primary treatment, but useful adjuvant therapies in aggressive prolactinomas can include radiation therapy and radiation therapy combined with temozolomide (TMZ) [25, 26]. Unfortunately, the efficacy of such regiments is variable, and significant side effects or complications may arise. Radiotherapy is recommended in clinically relevant tumor growth despite surgical resection in nonfunctioning tumors and after failed standard medical and surgical treatment in functioning tumors [25]. Additionally, adjuvant radiotherapy should be considered in the setting of a clinically relevant invasive tumor remnant with pathological markers (Ki-67 index, mitotic count, and p53 immunodetection) that strongly suggest aggressive behavior. All radiotherapy planning and discussion should occur under expert guidance with specific consideration for size, location, and prior radiotherapy dose. For patients with isolated metastases, consideration of loco-regional therapy should be considered independent of decisions regarding the need for systemic treatment [25]. The ESE is also recommended for patients with rapid tumor growth combining temozolomide with radiotherapy using the Stupp protocol. Overall management of these tumors requires a multidisciplinary approach.

## 4. Conclusion

Pituitary tumors are not uncommon. They represent a highly heterogeneous group varying histologically and in clinical relevance. They are routinely considered benign, but a surprising number can be locally invasive and up to 15% can be clinically aggressive. Pituitary tumors are classified as carcinomas when any anterior pituitary tumor cell line demonstrates distant metastasis or central nervous dissemination. Due to the rare incidence, the management of pituitary carcinoma is still very challenging and needs further investigation. Clinicians should suspect pituitary carcinoma with metastasis when there is a discrepancy between prolactin levels and MRI neuroimaging.
